# Beliefs of patients who visited community pharmacies about medicines and supplements, the need for drug therapy and medication adherence – a cross-sectional study

**DOI:** 10.1080/20523211.2024.2438235

**Published:** 2024-12-18

**Authors:** Etidal-Mihaela Manoliu-Hamwi, Cristina Gena Dascălu, Cristina Mihaela Ghiciuc, Georgeta Zegan, Elena-Mihaela Cărăușu, Mihaela Moscu, Cătălina Daniela Stan

**Affiliations:** a Department of Pharmaceutical Sciences II, Faculty of Pharmacy, “Grigore T. Popa” University of Medicine and Pharmacy, Iasi, Romania; b Department of Preventive Medicine and Interdisciplinarity, Faculty of Medicine, “Grigore T. Popa” University of Medicine and Pharmacy, Iasi, Romania; c Department of Morpho-Functional Sciences II, Faculty of Medicine, “Grigore T. Popa” University of Medicine and Pharmacy, Iasi, Romania; d St. Mary's Emergency Children Hospital, Iasi, Romania; e Department of Surgical, Faculty of Dental Medicine, “Grigore T. Popa” University of Medicine and Pharmacy, Iasi, Romania; f Department of Implantology, Removable Prosthesis, Denture Technology, Faculty of Dental Medicine, “Grigore T. Popa” University of Medicine and Pharmacy, Iasi, Romania

**Keywords:** Medication adherence, community pharmacists, beliefs about medicines, drug therapy, questionnaire, validity

## Abstract

**Background:**

Beliefs about medicines, the need for drug therapy and patient willingness can influence medication adherence. The community pharmacist, through his skills as an expert in drug therapy, has the opportunity to promote medication adherence in everyday pharmaceutical practice. This study aims to assess beliefs about medicines and supplements and the need for drug therapy and medication adherence of the patients who visited community pharmacies.

**Methods:**

809 patient volunteers answered a 14-item online questionnaire using a 5-point Likert scale. Statistical analysis was done in SPSS 27.0.

**Results:**

The validity indices of the questionnaire were acceptable; internal consistency was good; and the factor analysis indicated 3 factors (Eigen values > 1.0). The median scores were 3.33 regarding the beliefs about medicines and supplements; 2.75 regarding the beliefs about the need for therapy; and 2.71 on medication adherence. Beliefs about medicines and supplements, the need for drug therapy, and medication adherence varied according to patients’ socio-demographic status, health status, and access to pharmacy services (*p *≤ 0.05).

**Conclusion:**

Patients’ beliefs about medicines and supplements were moderate, patients’ beliefs about the need for drug therapy were negative and patients’ medication adherence was good.

## Background

The role of community pharmacists is changing from one that is focused on the supply of medicines and medical devices to one that is patient-centered, providing pharmacotherapy support. The pharmaceutical care as the clinical component, the quality of life of the patients as the humanistic component and the financial return of the pharmacy as the economic component are consequences of the quality of pharmaceutical services provided to patients. These components were the basis of the ECHO (Economic, Clinical and Humanistic Outcomes) model that used an integrated approach of pharmacoeconomic planning and evaluation of pharmaceutical services (Kozma et al., 1993). A key indicator in assessing the quality of patient care is ‘adherence to medication’, a concept introduced in the health system by the World Health Organization (WHO) (World Health Organization, 2015).

Adherence to medication indicates how well the patient adheres to the prescribed medication regimen and how long he remains under this treatment without interrupting it. The adherence threshold is 80% of doses taken versus the prescribed doses during treatment (Hugtenburg et al., 2013), but this threshold is arbitrary (Morrison et al, 2015; Baumgartner et al., 2018). On the other hand, medication non-adherence indicates non-application of the prescribed medication regimen regarding the number of doses and the duration of the treatment and is a complex public health problem. Medication non-adherence has clinical and economic implications, related to both negative therapeutic outcomes and mortality, as well as additional direct costs related to hospitalisations and indirect related to labour productivity. The WHO has estimated that approximately 50% of patients with chronic diseases are non-adherent to medication, with variations between countries (World Health Organization, 2003). The annual costs of medication non-adherence vary between continents, being €1.25 billion in Europe, up to US$290 billion in the USA and approximately $A7 billion in Australia (Cutler et al., 2018). Medication non-adherence has been studied by clinicians and health systems because of adverse outcomes and high health care costs (Ho et al., 2009).

Adherence to medication is determined by the interaction of several factors, which have been classified by the WHO into socio-economic factors, related to health personnel, health system, disease, therapy and patient (World Health Organization, 2003). A recent review by Folkvord et al. (2024) presented numerous studies in which these factors influenced the patients’ medication adherence. Patient beliefs and knowledge about medicines, need for drug therapy, and patient willingness influence medication adherence (Neame & Hammond, 2005; Castelan et al., 2023; Amdie et al., 2022).

The patient's motivational behaviour regarding medication non-adherence was classified by WHO into intentional and unintentional (World Health Organization, 2003). Intentional non-adherence to medication indicates non-application of the medication regimen prescribed by the deliberate decision of the patient (Hugtenburg et al., 2013; Patel, 2021). This may be due to various determinants, including side effects, medicines addiction, masking of other diseases, reduced long-term efficacy and patient stigma; but also to the perceptual barriers, such as lack of perceived need for drug therapy; and the practical barriers, such as access to medicines or treatment (Kardas et al., 2013). Unintended non-adherence to medication indicates unplanned patient behaviour such as forgetfulness, polypharmacy, low education level, poor understanding, mental illness, lack of confidence in prescribed treatment and other associated factors, e.g. material resources and demographic factors (Castelan et al., 2023).

The problem of medication non-adherence does not have a solution for all patients, but requires personalised interventions, depending on the causes (Castelan, et al., 2023). Physicians have well-known roles in promoting medication adherence. After physicians and nurses, the pharmacists are the third group of patient care professionals. The community pharmacists are in direct and regular contact with patients, being experts in drug therapy and skilled for education and counselling on medication adherence issues (Dillon et al., 2019; Faisal et al., 2021; Nind et al., 2022). Most community pharmacist-led interventions that manage medication adherence have been through indirect techniques, such as pharmacy dispensing records (Witry et al., 2018; Fenelon-Dimanche et al., 2020), pill counting, electronic pill counter, medication event monitoring system (Zijp et al., 2020; van Boven et al., 2021; Faisal et al., 2021; Morawski et al., 2018; Blakey et al., 2018; Nind et al., 2022; Ágh et al., 2024) or validated self-report adherence tools (Pednekar et al., 2019). Providing advanced guided counselling services to patients with chronic diseases was another community pharmacist-led intervention (van der Laan et al., 2017; Milosavljevic et al., 2018; Teeter et al., 2020; Jia et al., 2020; Daly et al., 2021; Shirdel et al., 2021; Torres-Robles et al., 2022; Smith-Ray et al., 2024). These pharmacist-led interventions have indicated varying degrees of success in behavioural, clinical, humanistic and economic outcomes (Pringle & Coley, 2015; Nazar et al., 2015; Stirrat et al., 2015; Folkvord et al., 2024).

Most medication adherence studies have been performed on patients with chronic diseases (Sjolander et al., 2013; Olorunfemi & Ojewole, 2018 Salama & Saudi, 2020). In the literature, there are few studies on the interventions of community pharmacists focused on advising patients for medication adherence, through preparing simple strategies for daily pharmaceutical practice (Boeni et al., 2015; Baumgartner et al., 2022). In Romania, only one study was conducted to evaluate medication adherence of chronic hepatitis C patients in a hospital, through a validated questionnaire (Turcu-Stiolica et al., 2021; Doica et al., 2023). Our study aims to assess beliefs about medicines and supplements, the need for drug therapy and adherence to medication of patients who benefited from the essential services of community pharmacists.

## Methods

### Study design

This cross-sectional study was conducted on patients in Romania who answered a questionnaire entered in Google Form, between January and June 2023.

The study had the following phases: (1) questionnaire development and validation, (2) assessment of patients’ beliefs about medicines and supplements, (3) assessment of patients’ beliefs about the need for drug therapy and (4) patients’ medication adherence.

### Study population

Only adult patients (over 18 years of age) who have previously benefited from essential services of community pharmacies such as medicines dispensing and recommendations for the correct administration were considered eligible. 1530 potential participants were deliberately recruited, using convenience sampling. Respondents were recruited through social media networks, such as email and WhatsApp, because in Romania there were restrictions for the population and health facilities, due to the Covid-19 pandemic from 2020 and 2023 (President of Romania, 2020; Romanian National Emergency Committee for Emergency Situations, 2023) and the flu epidemic from January 2023 and June 2023 (Romanian Ministry of Health, 2023a). Convenience sampling was based on volunteering and was conditioned by participants’ Internet access and digital skills. Respondents who completed the questionnaire selected themselves into the sample based on their accessibility and availability and distributed the questionnaire link to other difficult-to-identify possible participants, using snowball sampling to increase the size of the group. Sampling ended by saturation when no more data was recorded on the Google Form. The sample consists of 809 respondents, its size being adequate for the relevance of the results in this situational context.

### Study tool

We used a questionnaire in Romanian, consisting of 14 short, closed-ended and positive items, grouped into three domains regarding patients’ beliefs about (1) medicines and supplements, (2) the need for drug therapy, and (3) medication adherence.

Previous literature has provided several questionnaires, such as the ‘Beliefs about Medicines Questionnaire’ (BMQ) to assess patients’ beliefs and attitudes about medicines (Horne et al. 1999; Neame & Hammond, 2005), the ‘Purdue Pharmacist Directive Guidance Scale’ (PPDG) for measuring patients’ perceptions of the care received from pharmacists (Gupchup et al., 1996), the ‘Medication Adherence Reasons Scale’ (MAS-scale) (Unni et al., 2014) and the ‘Medication Adherence Rating Scale’ (MARS) for various aspects related to medication adherence (Horne & Weinman, 2002; Thompson et al., 2020). The expert group consisting of two pharmacists, a physician and academic staff specialised in pharmaceutical management, public health, clinical pharmacology, studied these questionnaires and considered none of these questionnaires to be in agreement with the objectives of our study. Therefore, the expert group developed a questionnaire of its own design in accordance with the intended purpose and the linguistic and cultural specificity and therefore it was not necessary to obtain permission from the original developers. The new questionnaire had a single version with 14 items.

The pilot version of the questionnaire was tested on a group of 25 participants, to assess the feasibility of using the questionnaire. The conveniently selected participants gave their verbal consent. 6 unclear items were subsequently revised by the panel of experts, keeping the 14 questions of the questionnaire.

The final version of the questionnaire was statistically analysed for content validity, reliability and internal structure regarding the grouping of the 14 items. The quality parameters of the questionnaire demonstrated through specific statistics will ensure the quality of the research results. The questionnaire is available on request.

Questionnaire response scale was a five-category Likert type, from 1 = strongly disagree to 5 = strongly agree. Patients’ beliefs about medicines and supplements and beliefs about the need for drug therapy were quantified by ‘negative beliefs’ (1 = strongly disagree and 2 = disagree), ‘moderate beliefs’ (3 = neutral) and ‘strong beliefs’ (4 = agree and 5 = strongly agree). Medication adherence was quantified by ‘adherent’ patients because they followed the prescription when they were ill (1 = strongly disagree and 2 = disagree); ‘partially adherent’ patients refers to the prescribed medication according to the circumstances of the presence or absence of the disease, meaning that when they were ill, they followed the prescription and when they were not ill they did not have any prescription (3 = neutral); and ‘non-adherent’ patients because they did not follow the prescription when they were ill (4 = agree and 5 = strongly agree).

The last section included patients’ socio-demographic status regarding gender, age, urban/rural environment, regions, education level, marital status, occupation and income; self-reported health status, member of a patient association, self-declared chronic patient and self-declared patient with co-morbidities; and access to pharmacy services regarding the frequency of visits to the pharmacy, the pharmacy visited and the way of purchasing medicines which represents patients’ experiences with community pharmacists who provided them with on demand pharmaceutical care in everyday practice.

### Statistical analysis

The validity of the questionnaire was statistically calculated with Inter-rater Agreement (IRA), Item Content Validity (ICV), Scale Content Validity (SCV), completeness index and reliability of the questionnaire sections with alpha-Cronbach coefficient. To evaluate the internal structure of the questionnaire, factor analysis was applied, using the ‘Principal axis factoring’ extraction method. Statistical processing was done in SPSS software (the Statistical Package for the Social Sciences) version 27.0 (SPSS Inc., Chicago, IL, USA).

The data collected automatically on the Google Form has been processed so that it can be interpreted. Respondents’ answers were coded from 1 to 5, depending on the answer given (1 = strongly disagree, 2 = disagree, 3 = neutral, 4 = agree and 5 = strongly agree). Based on them, the scores for the domains were calculated, by adding up the responses and were divided by the number of items in each domain. In this way, the variables corresponding to the answers of the questionnaire, which were originally ordinal, were transformed into continuous variables. We used the median values of the scores and the interquartile range (IQR) for the interpretation of the results, because the calculated scores did not follow the law of normal distribution (as determined by applying the Kolmogorov–Smirnov normal distribution fit test).

The median of the calculated scores were analysed compared to the respondents’ characteristics. Non-parametric tests (Mann–Whitney and Kruskall-Wallis) were used to reveal statistically significant differences between respondents. Statistically significant results were considered when *p* ≤ 0.05.

### Ethical considerations

The Ethics Commission of ‘Grigore T. Popa’ University of Medicine and Pharmacy Iasi approved this study (No. 237/19 November 2022). All participants gave their consent before voluntarily answering the online questionnaire, being informed about the purpose of the study and the anonymity of the data in a section preceding the questionnaire.

## Results

### Validation of the questionnaire

The group of evaluators evaluated the degree of clarity and relevance of each item of the questionnaire. Thus, the calculation of content validity items shows good values for IRA of 72% and ICV for of 84%. Very good values indicate SCV for clarity of 94%, for relevance of 90.3% and for completeness of 100%.

We calculated Cronbach's alpha coefficients showing an acceptable internal consistency of 0.795.

**Figure 1.**
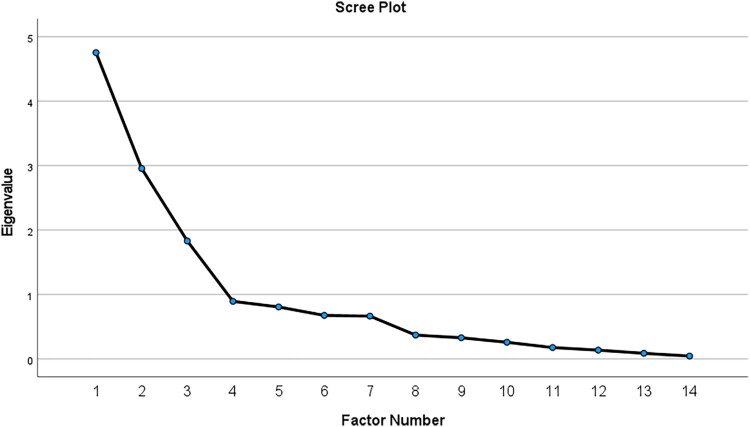
Scree plot of the investigated factors.

Factor analysis that detects the internal structure of the questionnaire indicates *p* = 0.000 for Bartlett's test of sphericity, which shows that the items are correlated with each other. The initial communalities of the questionnaire indicate high values, which show that the questions are well correlated, except for item 4, which has a low value (below 50%). The extracted communalities indicate high values (over 50%), which show that the items are covered by the factorial solution; items 1, 2, 4, 7 and 11 do not correlate well, having percentages below 50%. The factor analysis highlights 3 factors (with Eigen values > 1.0) responsible for 68.1% of the variation of the 14 investigated items, which represents a satisfactory percentage. The variance explained by the extracted factors drops to 60.2% after their rotation, recording a loss of approx. 8%, which is not covered by the model. From the factor matrix, the strongly correlated items and the other items with weak correlations below 0.2 are highlighted (Figure 1). Factor one is associated with items 8–14 and corresponds to the patients’ medication adherence section. Factor two is strongly associated with items 2, 5–7 and corresponds to the section of patients’ beliefs about the need for drug therapy. Factor three is moderately associated with items 1, 3 and 4 and corresponds to the section of patients’ beliefs about medicines and supplements.

The final version of the questionnaire meets the psychometric qualities for its validation.

### Characteristics of respondents

The valid response rate of the respondents was 100%, because the questionnaire had mandatory fields which did not allow the transition to another item and therefore there were no incomplete questionnaires.

809 patients enrolled in our sample, aged between 18 and 87 years and mean age 35.81 ± 13.52 (SD), of which 190 men with mean age 37.95 ± 14.63 (SD) and 619 women with mean age 35.16 ± 13.11 (SD). Most of the patients are from the urban environment (77.8%) and from the north-eastern region of Romania (77.3%). Most of the patients have university degrees (53.8%) and 65.6% are employees (Table 1).

**Table 1.** The socio-demographic status of the respondents.

Variables	N	%
**Gender**		
Male	190	23.5
Female	619	76.5
**Age group**		
18–20 years	57	7.0
21–30 years	301	37.2
31–40 years	188	23.2
41–50 years	147	18.2
51–60 years	73	9.0
Over 60 years	43	5.3
**Environment**		
Urban	629	77.8
Rural	180	22.2
**Regions**		
North-east	625	77.3
South-east	41	5.1
South	5	0.6
South-west	2	0.2
West	97	12.0
North-west	5	0.6
Center	10	1.2
Bucharest-Ilfov	24	3.0
**Educational level**		
Secondary	165	20.4
Undergraduate	435	53.8
Postgraduate	209	25.8
**Marital status**		
Married	374	46.2
Unmarried	380	47.8
Divorced	37	4.6
Widowed	18	2.2
**Occupation**		
Employed	531	65.6
Student	213	26.3
Retired	49	6.1
Unemployed	16	2.0
**Income**		
No income	172	21.3
Minimal income	92	11.4
Medium income	351	43.4
Above average income	194	24.0

**Figure 2.**
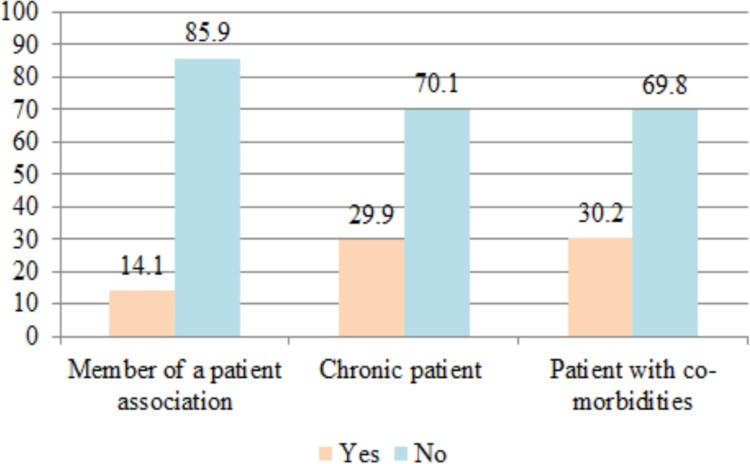
The self-reported health status of the respondents.

The self-reported health status of the respondents (on a scale from 1 to 10) is 8.29 ± 1.395 (SD), the majority declaring themselves without chronic diseases (70.1%) or co-morbidities (69.8%) (Figure 2). The most common chronic diseases of the patients were obesity (5.0%), hypertension (4.7%), diabetes (3.7%), autoimmune diseases (3.7%), other cardiovascular diseases (3.0%), digestive diseases (2.7%) and endocrine diseases (2.5%).

**Figure 3.**
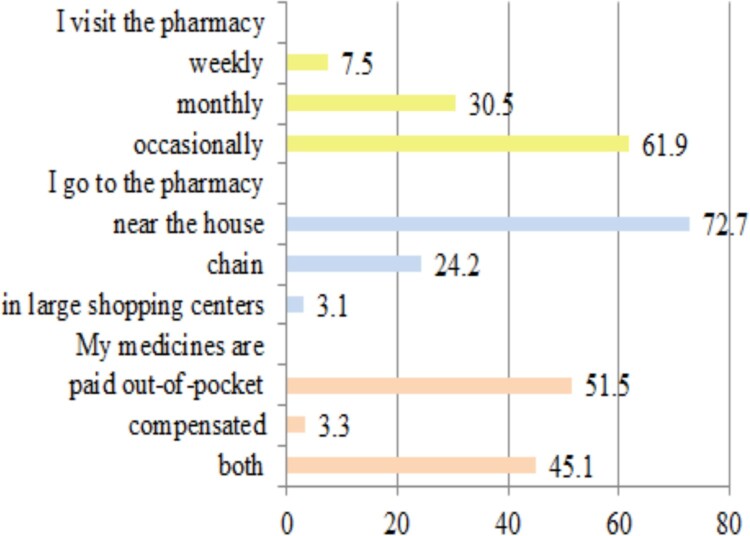
Respondents experiences with community pharmacies.

Respondents prefer to visit the pharmacies near their home (72.7%) to receive advice from community pharmacists, occasionally (61.9%) when they are sick, most often preferring to pay themselves for their own medicines (51.5%) (Figure 3).

### Assessment of patients’ beliefs about medicines and supplements

The median score of medicines and supplements beliefs is 3.33 and IQR 3.00 ÷ 4.00, which means that patients have moderate beliefs about of the importance of medicines and dietary supplements.

The median scores of beliefs about medicines and supplements show statistically significant differences according to gender (*p* = 0.000), age groups (*p* = 0.000), level of education (*p* = 0.021), marital status (*p* = 0.000), occupation (*p* = 0.001), income (*p* = 0.007) (Table 2); membership of a patient association (*p* = 0.003), own declared chronic patient (*p* = 0.000), own declared patient with co-morbidities (*p *= 0.000) (Table 3); and the pharmacy visited (*p* = 0.045) (Table 4).

**Table 2.** Comparison of scores according to the patients' socio-demographic status.

Socio-demographic status	Beliefs about medicines and supplements			The need for drug therapy			Medication adherence		
	Median	IQR	*P*-value	Median	IQR	*P*-value	Median	IQR	*P*-value
**Gender**			0.000*			0.525			0.088
Male	3.33	3.00 ÷ 3.66		2.75	2.00 ÷ 3.75		2.57	2.14 ÷ 3.14	
Female	3.66	3.33 ÷ 4.00		2.75	2.25 ÷ 3.50		2.71	2.28 ÷ 3.14	
**Age group**			0.000*			0.000*			0.346
18–20 years	3.33	3.00 ÷ 3.66		2.50	1.75 ÷ 3.00		2.85	2.28 ÷ 3.14	
21–30 years	3.66	3.33 ÷ 4.00		2.50	2.00 ÷ 3.25		2.71	2.14 ÷ 3.14	
31–40 years	3.66	3.00 ÷ 4.00		2.75	2.00 ÷ 3.25		2.85	2.28 ÷ 3.28	
41–50 years	3.33	3.00 ÷ 3.66		3.00	2.50 ÷ 3.75		2.71	2.28 ÷ 3.14	
51–60 years	3.33	3.00 ÷ 3.66		3.75	3.00 ÷ 4.25		2.57	2.21 ÷ 3.00	
Over 60 years	3.33	3.00 ÷ 3.66		4.00	3.50 ÷ 4.25		2.71	2.28 ÷ 3.00	
**Environment**			0.713			0.960			0.043*
Urban	3.66	3.00 ÷ 4.00		2.75	2.25 ÷ 3.50		2.71	2.14 ÷ 3.14	
Rural	3.33	3.00 ÷ 3.66		2.75	2.25 ÷ 3.50		2.85	2.28 ÷ 3.28	
**Regions**			0.143			0.047*			0.099
North-east	3.66	3.33 ÷ 4.00		2.75	2.00 ÷ 3.50		2.71	2.28 ÷ 3.14	
The rest of the country	3.33	3.00 ÷ 3.66		3.00	2.25 ÷ 3.75		2.64	2.14 ÷ 3.00	
**Educational level**			0.021*			0.004*			0.000*
Secondary	3.33	3.16 ÷ 3.66		3.25	2.25 ÷ 3.75		3.00	2.42 ÷ 3.28	
Undergraduate	3.66	3.33 ÷ 4.00		2.75	2.25 ÷ 3.50		2.71	2.14 ÷ 3.14	
Postgraduate	3.33	3.00 ÷ 3.66		2.75	2.00 ÷ 3.50		2.71	2.14 ÷ 3.14	
**Marital status**			0.000*			0.000*			0.643
Married	3.33	3.00 ÷ 3.66		3.00	2.25 ÷ 3.75		2.71	2.28 ÷ 3.14	
Unmarried	3.66	3.33 ÷ 4.00		2.50	2.00 ÷ 3.25		2.71	2.14 ÷ 3.14	
Divorced	3.66	3.00 ÷ 3.83		3.25	1.87 ÷ 4.00		2.85	2.21 ÷ 3.28	
Widowed	3.33	3.00 ÷ 3.66		4.00	3.68 ÷ 4.56		2.85	2.28 ÷ 3.28	
**Occupation**			0.001*			0.000*			0.814
Employed	3.33	3.00 ÷ 3.66		2.75	2.25 ÷ 3.50		2.71	2.14 ÷ 3.14	
Student	3.66	3.33 ÷ 4.00		2.50	2.00 ÷ 3.00		2.71	2.28 ÷ 3.14	
Retired	3.33	3.00 ÷ 3.66		4.00	3.50 ÷ 4.37		2.57	2.28 ÷ 3.00	
Unemployed	3.50	2.83 ÷ 3.91		2.75	1.81 ÷ 3.75		2.71	2.25 ÷ 3.28	
**Income**			0.007*			0.000*			0.000*
No income	3.66	3.33 ÷ 4.00		2.50	2.00 ÷ 3.18		2.78	2.14 ÷ 3.14	
Minimal income	3.33	3.00 ÷ 3.66		3.00	2.25 ÷ 3.75		2.85	2.42 ÷ 3.28	
Medium income	3.66	3.33 ÷ 4.00		3.00	2.25 ÷ 3.75		2.71	2.28 ÷ 3.14	
Above average income	3.33	3.00 ÷ 3.66		2.75	2.00 ÷ 3.75		2.42	2.00 ÷ 3.00	

Mann-Whitney and Kruskall-Wallis tests.

*Significant level for differences at (*p* ≤ 0.05).

**Table 3.** Comparison of scores according to the self-reported patients’ health status.

Health status	Beliefs about medicines and supplements			The need for drug therapy			Medication adherence		
	Median	IQR	*P*-value	Median	IQR	*P*-value	Median	IQR	*P*-value
**Patient associations**	0.003*		0.003*			0.068			0.411
Yes	3.66	3.33 ÷ 4.00		3.00	2.25 ÷ 3.50		2.71	2.14 ÷ 3.28	
No	3.33	3.00 ÷ 3.66		2.75	2.25 ÷ 3.50		2.71	2.14 ÷ 3.14	
**Chronic patient**			0.000*			0.000*			0.500
Yes	3.66	3.00 ÷ 3.66		3.75	2.75 ÷ 4.00		2.71	2.14 ÷ 3.14	
No	3.33	3.33 ÷ 4.00		2.50	2.00 ÷ 3.25		2.71	2.28 ÷ 3.14	
**Co-morbidities patient**			0.000*			0.000*			0.505
Yes	3.33	3.00 ÷ 3.66		2.75	2.75 ÷ 4.00		2.71	2.14 ÷ 3.14	
No	3.66	3.33 ÷ 4.00		2.50	2.00 ÷ 3.25		2.71	2.28 ÷ 3.14	

Mann-Whitney and Kruskall-Wallis tests.

*Significant level for differences at (*p* ≤ 0.05).

**Table 4.** Comparison of scores according to the pharmacies visited and the medicines purchased.

Accessing pharmacy services	Beliefs about medicines and supplements			The need for drug therapy			Medication adherence		
	Median	IQR	*P*-value	Median	IQR	*P*-value	Median	IQR	*P*-value
**I visit the pharmacy**			0.176			0.000*			0.721
Weekly	3.33	3.00 ÷ 4.00		3.25	2.75 ÷ 4.00		2.71	2.28 ÷ 3.28	
Monthly	3.33	3.00 ÷ 3.66		3.50	2.50 ÷ 4.00		2.71	2.14 ÷ 3.00	
Occasionally	3.66	3.33 ÷ 4.00		2.50	2.00 ÷ 3.25		2.71	2.14 ÷ 3.14	
**I go to the pharmacy**			0.045*			0.019*			0.716
Near the house	3.33	3.00 ÷ 3.66		2.75	2.00 ÷ 3.50		2.71	2.14 ÷ 3.14	
Chain	3.66	3.33 ÷ 4.00		3.00	2.25 ÷ 3.75		2.71	2.28 ÷ 3.14	
In large shopping centres	3.33	3.00 ÷ 3.38		2.75	2.12 ÷ 3.75		2.71	2.42 ÷ 3.14	
**My medicines are**			0.071			0.000*			0.761
Paid out-of-pocket	3.66	3.00 ÷ 4.00		2.50	2.00 ÷ 3.00		2.71	2.14 ÷ 3.14	
Compensated	3.33	3.00 ÷ 3.66		3.50	3.25 ÷ 4.00		2.57	2.28 ÷ 3.00	
Both	3.33	3.00 ÷ 4.00		3.25	2.50 ÷ 4.00		2.71	2.21 ÷ 3.14	

Mann-Whitney and Kruskall-Wallis tests.

*Significant level for differences at (*p* ≤ 0.05).

### Assessment of patients’ beliefs about the need for drug therapy

Beliefs about the need for drug therapy have a median of 2.75 and IQR 2.25 ÷ 3.50, meaning that patients are not convinced that they need drug therapy.

The median scores of beliefs about the need for drug therapy show statistically significant differences according to age groups (*p* = 0.000), regions (*p* = 0.047), education level (*p* = 0.004), marital status (*p* = 0.000), occupation (*p* = 0.000), income (*p* = 0.000) (Table 2), own declared chronic patient (*p* = 0.000), own declared patient with co-morbidities (*p* = 0.000) (Table 3), frequency of visits to the pharmacy (*p* = 0.000), the frequented pharmacy (*p* = 0.019) and the method of purchasing medicines (*p* = 0.000) (Table 4).

The median score is 4.00 in the case of patients over 60 years old, divorced and retired, which means that they were convinced of the need for drug therapy (Table 2).

### Assessment of patients’ medication adherence

The scores of the intentional and unintentional reasons of this domain of the questionnaire have median of 2.71 and IQR 2.14 ÷ 3.14, which means that patients are adherent to the medication.

The median scores of medication adherence show statistically significant differences according to urban/rural area (*p* = 0.043), education level (*p* = 0.000) and income (*p* = 0.000) (Table 2).

## Discussion

In this study, we identified the beliefs about medicines and supplements, the need for drug therapy, and adherence to medication of patients who accessed the essential services of community pharmacies and were accessible and available on the online platforms, specific to the restrictions during the recent pandemic and epidemic in Romania. In the Covid-19 pandemic there was an exodus of confirmed cases and deaths worldwide. In Romania, until the end of June 2023 when our survey was also done, there were 3,402,356 confirmed cases of Covid-19 infections and 68,172 deaths (Romanian National Institute of Public Health, 2023). Also, the epidemic flu with influenza virus, coronavirus and respiratory syncytial virus that existed in the first half of 2023 in Romania registered 3900 confirmed cases and 93 deaths (Romanian Ministry of Health, 2023b). Patients with these infectious diseases have overburdened medical and pharmaceutical services (Wong et al., 2023), being the largest consumers of medicines compared to patients with chronic diseases. Thus, another perspective has opened up on diseases and medication adherence.

We developed a new questionnaire to measure patients’ opinions about medicines and supplements, the need for drug therapy and medication adherence. The questionnaire required validation for our results to be sustainable.

### Patients’ beliefs about medicines and supplements

In our study, patients’ beliefs about taking medicines in case of illness, daily intake of dietary supplements for a nutrient-balanced diet, as well as compliance with the pharmacist's recommendations were moderate. The advice provided by the pharmacists was probably not entirely convincing for some reason. In Romanian pharmaceutical practice, the number of pharmacists is insufficient, and pharmacy assistants take over a part of the tasks. Also, the workload is high and consequently the time given to patients is too short. Community pharmacy managers should remedy these problems through appropriate measures based on the skills of the pharmacy staff.

Neame and Hammond (2005) showed that beliefs about medicines are dynamic and can change with relevant and updated information. In general, knowledge about medicines includes the name, indication, way of administration, side effects and some dietary restrictions, which is obtained from the leaflet, from general practitioners or specialists, pharmacists or by searching the Internet. This knowledge informs the patient about his medications, influencing his behaviour in their administration, confidence in their usefulness and can be associated with intentional non-adherence to medication (Zeber et al., 2013; Mekonnom & Gelayee, 2020; Castelan et al., 2023).

During the Covid-19 pandemic, in Romania there were numerous advertisements in the media about dietary supplements that could be found in community pharmacies and were requested by patients. Su et al. (2024) found moderate compliance with dietary supplements for postoperative digestive tract tumours patients in Shanghai that varied with age, education level, adverse reactions, medicines beliefs, social support and self-efficacy.

Informing the patient about medicines or supplements is not educating the patient. Community pharmacists are most skilled in counselling and educating patients about drug therapy. An advanced consultation service for patient education in this regard would be necessary to implement in Romania.

In our study, beliefs about medicines and supplements varied with some socio-demographic features such as gender, age, education level, marital status, occupation and income. Pharmacists’ counselling should be made according to patients’ gender, age and marital status so that they are convinced that the recommendations they receive are appropriate. The pharmacist's language during counselling should be adapted to the patients’ level of education in order to understand the explanations. Occupation and income reflected patients’ standard of living and influenced the power of medicines purchasing. Chapdelaine et al. (2021) assessed socio-demographic factors and beliefs about medicines on pharmacogenomic testing and they reported that lower than university education was associated with not accepting payment for these tests.

In our study, beliefs about medicines and supplements were influenced by patient association membership and patients’ chronic diseases and co-morbidities. In the study group, half of the patients with chronic diseases were members of a patient association. In Romania there are 235 patient associations that offer free guidance, counselling and information and defend their constitutional rights to health (Romanian National Authority for Quality Management in Health, 2024). One third of the patients of the study group presented chronic diseases and co-morbidities, in agreement with the self-reported data of the population in 2023, in a national health status report (European Commission, 2023).

Previous studies showed that medication adherence of chronic diseases patients was related to their beliefs about medicines. Sjolander et al. (2013) studying hospitalised stroke patients in Sweden reported that adherents to medication had positive beliefs about medicines and non-adherents had negative beliefs, mostly men. Salama and Saudi (2020) reported that half of diabetic patients in a family medicine clinic in Egypt were non-adherent to medication due to beliefs about medicines regarding the concerns about the side-effects, being influenced by age and education.

In our study, beliefs about medicines and supplements were influenced by the pharmacy attended. Most respondents of the study group went to the pharmacy closest to their home to benefit from the services of pharmacists. Probably this behaviour of the majority of the respondents was attributed to the pandemic and epidemiological context in Romania which generated a social anxiety (Leichsenring & Lewekw, 2017) towards the disease, and towards the restrictions imposed to reduce the spread of viruses (Chandola et al., 2022; Wang et al., 2023).

### Patients’ beliefs about the need for drug therapy

In our study, patients’ beliefs about the need for drug therapy in the event of the onset or worsening of the disease, as well as the return of expired medicines to the pharmacy, were negative. Presumably, at the time of the survey, the respondents were in good health. In the year 2022 in a country report, 73.3% of Romanians reported that their state of health is very good or good, exceeding the European Union average of 68.0% (European Commission, 2023).

In the Romanian health system, community pharmacists deliver medicines based on medical prescriptions, over-the-counter (OTC) medicines and supplements (Romanian Parliament, 2015). Usually, the patient waits in the pharmacy with the compensated prescription and the health card until it is his turn at the counter. The pharmacist can deliver the original or generic medicines of choice, depending on the patient's budget. The medical prescription can contain a maximum of seven compensated medicines from the lists established by the Ministry of Health (Romanian National Health Insurance House, 2024b). The pharmacist performs several procedures until the medicines are delivered, such as prescription verification, pharmaceutical and pharmacological evaluation of the prescription, and verification of supply resources. The pharmacist informs the patient about the way of administration, side effects and interactions between the medicines, with alcohol or with some foods (Romanian College of Pharmacists, 2021). In the therapeutic relationship between pharmacist and patient, the pharmacist ensures that the patient has understood the recommendations and the patient becomes responsible for medication adherence. During the weekend, when the physician is not available, when the patient does not have a medical prescription or is from another city and does not have the medicines with him, the pharmacist can give a single dose of the requested medicine, if he considers it to be a medical emergency (Romanian Ministry of Health, 2024b). This measure prevents inappropriate, uncontrolled and excessive use of medicines to ensure patient safety.

During the Covid-19 pandemic in Romania, there was no treatment protocol for the SARS-CoV-2 virus, except for hospitalised patients. Patients with the first symptoms of the disease turned to telepharmacy due to discontinuities in medical services. Pharmacists counselled the patients by phone and recommended a quick at-home Covid-19 antigen test. In the case of a positive test, patients were to self-isolate and call the emergency system and their family physician to request medical assistance. The patient received the prescription from the family physician on WhatsApp or email and submitted it to the pharmacy, and a family member collected the medicines and administration recommendations from the pharmacists (Romanian College of Pharmacists, 2020). Patients with influenza symptoms from the recent epidemic period were treated symptomatically with OTCs and were quarantined (Romanian National Institute of Public Health, 2022). The global public health emergency caused by the Covid-19 virus has placed pharmacists in the front line of intervention in patient health care (Bukhari et al., 2020; Hedima et al., 2020).

During the pandemic and epidemic period in Romania, antiviral medicines were missing from community pharmacies and patients with financial possibilities bought these medicines from neighbouring countries. To remedy these discontinuities in the supply of medicines to the population, there should be a permanent collaboration between the Romanian Ministry of Health and the European Medicines Agency.

This section had an item about expired medications, because some patients tend to stock up on medications. Expired medicines are considered hazardous waste for the environment, because they pollute and contaminate the soil and waters and are harmful factors for the health of human life. Until recently, patients took unused or expired medicines to community pharmacies to be collected and then handed over and neutralised by other companies (Romanian Ministry of Health, 2014; 2018). Most patients do not know what to do with these medicines and usually throw them in the garbage bin. More recently, this collection procedure has become more difficult for patients, because the hospitals must collect expired medicines (Romanian Ministry of Health, 2024a).

Some patients’ characteristics such as age, location on regions, level education, marital status, occupation and income, influenced patients’ beliefs about to the need for drug therapy in our study. Patients’ age could be a barrier to their beliefs about the need for drug therapy. Access to the pharmacy could be limited by the patient's location in different regions and the distance they have to travel to the pharmacy. Understanding the need for drug therapy depends on the level of education and the income has allows him to buy or not the necessary medicines.

In our study, beliefs about the need for drug therapy were influenced by patients’ chronic diseases and co-morbidities. Only patients over 60 years old, retired and divorced respondents required drug therapy, being probably patients with chronic diseases and co-morbidities. Studies focusing on patients with various diseases have reported positive attitudes towards the need for prescription medicines (Agrawal et al., 2023; Barry et al., 2023; Koltuniuk & Chojdak-Koltuniuk, 2023; Sipos et al., 2021). Thorneloe et al. (2017) recommended the introduction of specific items for the diseases of the interviewed patients, in order to accurately identify their beliefs about the prescribed medication regimens. During the Covid-19 pandemic, Romanian pharmacists strictly followed the treatment of chronic patients in order not to decompensate. In the situation where chronic patients with mild symptoms of SARS-CoV-2 virus infection requested the services of community pharmacies, the pharmacists monitored some biological parameters and directed the patients to emergency departments (Romanian College of Pharmacists, 2020).

In our study, patients’ beliefs about the need for drug therapy were influenced the frequency of visits to the pharmacy, the pharmacy visited and the way of purchasing medicines. Most of the respondents used pharmaceutical services occasionally, probably when they needed medicine for their illness. Most of the respondents went to the pharmacy near their home, which means they asked the pharmacists they usually go to for advice. Most of the respondents paid for the medicines, which means that they did not go to the family physician for a compensated prescription to cover the costs of the medicines through the social health insurance system, but went to the pharmacist first. Community pharmacists have a considerable role in the relational continuity of pharmaceutical care and patients who visited a single pharmacy had better medication adherence than those who visited multiple pharmacies (Choi & Lee, 2022).

Hwang et al. (2024) showed that there is a relationship between the need for drug therapy and beliefs about medicines. Thus, studying cancer patients with oral anticancer therapies found that some of them had positive attitudes and others had negative attitudes towards the medicines. Previous studies have shown that there is a link between medication adherence and beliefs about the need for drug therapy in patients with various diseases (Cea-Calvo et al., 2020; Castelan et al., 2023). Brett et al. (2018) studying women with adjuvant endocrine therapy after breast cancer in the U.K. found that only a quarter of women were non-adherent to medication, and that side effects, younger age and their post-secondary education were associated. Swiatoniowska-Lonc et al. (2021) studying hospitalised hypertensive patients in Poland found that intentional nonadherence to medication was moderate, being influenced by concerns about medicines side effects, age, co-morbidity, and loneliness.

### Medication adherence

In order to identify patients’ beliefs about their medication adherence, many items were used with intentional reasons such as, alternatives therapies holistic or bioenergetics using, concerns about daily administration, long-term unwanted effects and treatment interruption; and with unintentional reasons such as, difficulty of daily administration, forgetfulness and lack of money. Scores indicated that patients were adherent to medication, with the pandemic and epidemic context likely contributing. Our results were similar to a study conducted in a hospital in Romania, on patients with antiviral treatments for hepatitis C, which reported improvements in medication adherence and Health-Related Quality of Life (Doica et al., 2023). Studies focusing on patients with certain diseases have reported various degrees of medication adherence, from poor to high (Dessie et al., 2020; Li et al., 2023; Alsubaie et al., 2024). Studies of pharmacist-led interventions for patients with chronic diseases have indicated improved medication adherence (Alfian et al., 2021; Zheng et al., 2022; Wu et al., 2023). Konstantinou et al. (2022) in a literature review on methods for assessing medication adherence of patients with chronic diseases, recommended the combination between self-report like MARS-5 and non-self-report measures like MEMS.

In our study, patient responses varied with some socio-demographic features such as, urban/rural environment, education level and income, which may be interpreted as barriers to medication adherence. Medication adherence barriers can be related to the patient, the service provider or the health system (Jimy & Jose, 2011; Silva et al., 2022; Fenta et al., 2024). In the health system in Romania, the number of community pharmacies and pharmacists in the rural environment is lower than in the urban environment, although the population in the rural environment is approximately equal to that in the urban environment (Romanian National Institute of Statistics, 2022). Probably because of this, the quality of counselling for rural patients constituted a barrier for the medication adherence of these patients. In rural areas, 70% of the inhabitants are agricultural workers (Romanian National Institute of Statistics, 2022). It is likely that the group of patients with a low level of education have had difficulties in understanding the pharmacists’ recommendations, which represented a barrier to medication adherence. Also, during the Covid-19 pandemic, communication with pharmacists was possible through e-Health (Romanian Parliament, 2018), and patients without online skills acquired through education did not have access. The group of patients with limited incomes was affected by economic access to medicines, because the cost of medicines was continuously increasing (Romanian Ministry of Health, 2022), which could be a cause of unintentional non-adherence to medication. For some categories of patients, the social health insurance system in Romania fully or partially covered many generic medicines (Romanian National Health Insurance House, 2024a).

Previous studies have reported many barriers that influenced medication adherence (Kelly et al., 2014; Chan et al., 2021). The interaction of factors related to the patient, their disease characteristics, social contexts and access to pharmaceutical services indicated the complexity of medication adherence (Wu et al., 2023). Castelan et al. (2023) showed that young HIV patients were non-adherent to prescribed treatment and ethnic minorities had high levels of stigma, depressive symptoms, language barriers and low social support. Kubica et al. (2017) showed that the medication adherence of patients with coronary heart disease after myocardial infarction was influenced by the health status and age of the patients, number of hospitalisations and the association of the disease with diabetes mellitus. Previous studies of patients with chronic disease have shown that social and environmental factors, such as social interaction and support networks and economic factors, have significant effects on medication adherence (Park et al., 2015; Wooldridge et al., 2015; Zullig et al., 2015; Reddy et al., 2017; Shankari et al., 2020).

Our study contributes to medication adherence related to beliefs about medicines and supplements and the need for drug therapy of patients who benefited from essential services of community pharmacists, during pandemic and epidemic periods, being the only study in the literature as far as we know. In parallel with the previous literature, medication adherence was studied on patients with chronic diseases, outside the period of the Covid-19 pandemic, with physicians and pharmacists being trained to address strategies to improve medication adherence.

Our study has practical applicability for improving the quality of the pharmaceutical care of the community pharmacists, through counselling strategies focused on the unique characteristics of each patient, to increase patient loyalty and actively contribute with the medical team to medication adherence. The professional perspective of the medical team should be oriented towards maintaining health through multidisciplinary prevention programmes based on educational and cognitive–behavioural interventions. In the future, it would be useful to introduce consultation advanced services (Romanian Ministry of Health, 2021) for medication adherence by community pharmacy managers. Also, it would be useful to introduce new digital technologies to monitor patients’ medication adherence (Silva et al., 2022), so that pharmacists have new perspectives to prevent medication non-adherence.

Our study presents a few limitations. Firstly, the recruitment of participants was done on social media platforms, due to the reduction of human interactions imposed by the Covid-19 pandemic. Thus, certain categories of participants, such as those living in areas with limited Internet access, those without digital technologies, or some elderly people who do not use social networks, could not participate in this study.

Secondly, convenience and snowball sampling were based on self-selection of accessible and available respondents from online platforms, specific to pandemic restrictions. Convenience sampling is easy and quick, but it is not representative of a population. Snowball sampling is used for hard-to-reach people and is based on known people of existing participants, usually with the same characteristics, which could affect the heterogeneity of the group. Therefore, the sample was representative for women from the urban environment and from the north-eastern region of Romania, with university education, employed and with online skills. Sampling methods may have influenced the study results, which are relevant to this pandemic and epidemic crisis context and cannot be generalised to other future contexts. It would be necessary to repeat this study outside of pandemic and epidemic circumstances, using stratified probability sampling, to compare the results.

Thirdly, due to the cross-sectional design of our study no causal associations were made between the dependent and independent variables, but the relatively large sample size provided confidence in the significance of our findings. Our study can be continued by analysing preconditions among independent variables, to assess the strength of certain factors in medication adherence, which are not yet studied.

Fourthly, the self-report assessment method may lead to overestimation of responses and our results should be interpreted with caution, although questionnaires are easy to process and remain the best valid instruments.

## Conclusion

Our questionnaire that assesses beliefs about medicines and supplements, the need for drug therapy and medication adherence of patients who visited community pharmacies was developed and validated. Patients’ beliefs about medicines and supplements were moderated and were influenced by some socio-demographic characteristics, health status and pharmacy attended. Patients’ beliefs about the need for drug therapy were negative and they varied according to some socio-demographic characteristics, health status and access to community pharmacy services. Patients over 60 years old, retired or divorced had positive beliefs about drug therapy. Patients’ medication adherence was good, being influenced by urban/rural environment, education level and income.

## Data Availability

The datasets used and analysed during the current study are available from the corresponding author on reasonable request. The questionnaire may be available after the defence of the doctoral thesis.
